# Restricted DCJ-indel model: sorting linear genomes with DCJ and indels

**DOI:** 10.1186/1471-2105-13-S19-S14

**Published:** 2012-12-19

**Authors:** Poly H da Silva, Raphael Machado, Simone Dantas, Marília DV Braga

**Affiliations:** 1 IME, Universidade Federal Fluminense, Niterói, Brazil; 2 Inmetro - Instituto Nacional de Metrologia, Qualidade e Tecnologia, Duque de Caxias, 25250-020, Brazil

## Abstract

**Background:**

The double-cut-and-join (DCJ) is a model that is able to efficiently sort a genome into another, generalizing the typical mutations (inversions, fusions, fissions, translocations) to which genomes are subject, but allowing the existence of circular chromosomes at the intermediate steps. In the general model many circular chromosomes can coexist in some intermediate step. However, when the compared genomes are linear, it is more plausible to use the so-called restricted DCJ model, in which we proceed the reincorporation of a circular chromosome immediately after its creation. These two consecutive DCJ operations, which create and reincorporate a circular chromosome, mimic a transposition or a block-interchange. When the compared genomes have the same content, it is known that the genomic distance for the restricted DCJ model is the same as the distance for the general model. If the genomes have unequal contents, in addition to DCJ it is necessary to consider indels, which are insertions and deletions of DNA segments. Linear time algorithms were proposed to compute the distance and to find a sorting scenario in a general, unrestricted DCJ-indel model that considers DCJ and indels.

**Results:**

In the present work we consider the restricted DCJ-indel model for sorting linear genomes with unequal contents. We allow DCJ operations and indels with the following constraint: if a circular chromosome is created by a DCJ, it has to be reincorporated in the next step (no other DCJ or indel can be applied between the creation and the reincorporation of a circular chromosome). We then develop a sorting algorithm and give a tight upper bound for the restricted DCJ-indel distance.

**Conclusions:**

We have given a tight upper bound for the restricted DCJ-indel distance. The question whether this bound can be reduced so that both the general and the restricted DCJ-indel distances are equal remains open.

## Background

The distance between two genomes is often computed using only the common content, which occurs in both genomes. Such distance takes into consideration only *organizational *operations, which change the organization of the genome, that is, the positions and orientations of DNA segments, number and types of chromosomes. Inversions, translocations, fusions and fissions are some of these operations [1]. All these rearrangements can be generically represented as *double-cut-and-join *(DCJ) operations [2]. The DCJ model has simple linear algorithms to compute the distance and to find an optimal sorting sequence [3]. However, while sorting a genome into another by DCJ, circular chromosomes can appear in the intermediate species [3]. In the *general model *many circular chromosomes can coexist in some intermediate species. Due to this fact, when the compared genomes are linear, it is desirable to consider the so-called *restricted model*, in which we proceed the reincorporation of a circular chromosome immediately after its creation [2,4]. These two consecutive DCJ operations, which create and reincorporate a circular chromosome, mimic a transposition or a block-interchange. In other words, in the restricted model most of the classical organizational operations (reversals, translocations, fusions and fissions) cost one DCJ, while transpositions and block-interchanges cost two DCJs.

**Figure 1:**
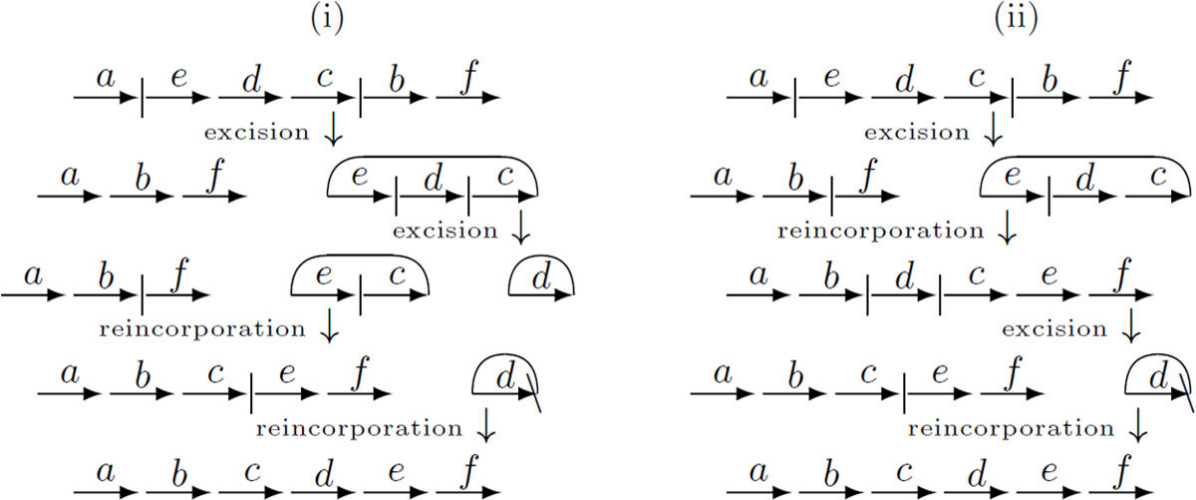
**(i) An optimal sorting sequence in the general DCJ model - many circular chromosomes can coexist in the intermediate species**. (ii) An optimal sorting sequence in the restricted DCJ model - a circular chromosome is immediately reincorporated after its excision. The distance is always the same for both general and restricted DCJ models.

When comparing genomes with the same content and without duplicated DNA segments, it is already known that the genomic distance for the restricted DCJ model is the same as the distance for the general model and can be computed in linear time [2,3]. In contrast, while the genomes can be sorted also in linear time in the general model [3], the best sorting algorithm in the restricted model up to now takes *O*(*n *log *n*) [4]. Figure 1 shows an example of a general and a restricted sorting sequence.

If the genomes have unequal contents, in addition to DCJ operations it is necessary to consider *insertions *and *deletions *of DNA segments. Insertions and deletions are jointly called indels. In this context, linear algorithms were proposed to compute the distance and to find a sorting scenario in a general, unrestricted model that handles genomes without duplicated DNA segments, considering DCJ and indel operations [5,6]. During the evolution of many organisms, indel operations are said to occur more often than organizational operations and, consequently, should be assigned to a lower cost. Examples are bacteria that are obligate intracellular parasites, such as *Rickettsia *[7]. The genomes of such intracellular parasites are observed to have a reductive evolution, that is, the process by which genomes shrink and undergo extreme levels of gene degradation and loss.

**Figure 2:**
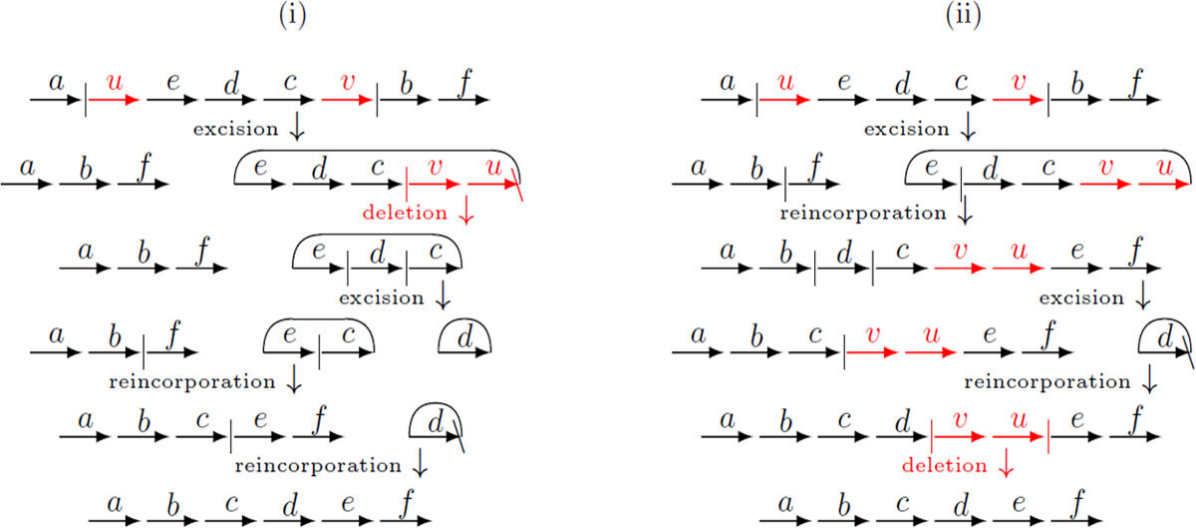
**(i) An optimal sorting sequence in the general DCJ-indel model - many circular chromosomes can coexist in the intermediate species**. (ii) An optimal sorting sequence in the restricted DCJ-indel model - a circular chromosome is immediately reincorporated after its excision. Although the number of steps in (i) and (ii) is the same, the question whether the distance is the same for both general and restricted DCJ-indel models is open. (The common content of the initial and the final genomes is represented in black, while the content exclusive to the initial genome is represented in red.).

The general DCJ-indel model has the flexibility of assigning different positive costs to DCJ and indel operations [5,6]. But, again, many circular chromosomes may coexist in intermediate stages of the sorting process. Thus, while sorting linear genomes, it would be more plausible to consider a restricted DCJ-indel model, in which a circular chromosome must be reincorporated immediately after its creation. Figure 2 shows an example of a general and a restricted sorting sequence with DCJs and indels. In this case, no algorithm was provided up to now and even the question whether the distance is the same for both the general and the restricted DCJ-indel models remains open. Here we address this issue and give a sorting algorithm and a tight upper bound for the restricted DCJ-indel distance, also allowing the assignment of distinct costs to indel and DCJ operations and with the restriction that the indel cost is upper bounded by the DCJ cost.

This paper is organized as follows. In the remainder of this section we recall some key concepts of the DCJ-indel model with distinct operation costs [6], which is based on the DCJ-model [2,3]. We then develop a restricted DCJ-indel sorting algorithm, that gives an upper bound for the restricted DCJ-indel distance. Finally we conclude our work with some final remarks.

### The DCJ model

A linear genome is composed of linear chromosomes and can be represented by a set of strings as follows. For each chromosome  C of each genome, we build a string obtained by the concatenation of all markers in  C. Each marker *g *is a DNA fragment and is represented by the symbol *g*, if it is read in direct orientation, or by the symbol  ḡ, if it is read in reverse orientation. Each one of the two extremities of a linear chromosome is called a *telomere*, represented by the symbol ○.

Given two linear genomes *A *and *B*, possibly with unequal content, let  G,  A and  B be three disjoint sets, such that the set  G is the set of markers which occur in *A *and in *B*, the set  A is the set of markers which occur only in *A *and the set  B is the set of markers which occur only in *B*. The markers in  A and in  B are also called *unique markers*. As an example, consider the genomes $A=\left\{\circ bsu\bar{c}av\bar{d}e\circ \right\}$ and $B=\left\{\circ awb\bar{x}c\circ ,\circ ydze\circ \right\}$. Here we have G={a,b,c,d,e}, A={s,u,v} and B={w,x,y,z}.

Given two genomes *A *and *B*, we denote the two extremities of each g∈G by *g^t ^*(tail) and *g^h ^*(head). A  G-adjacency or simply adjacency [5] in genome *A *(respectively in genome *B*) is a string $v=\gamma_{1}\ell \gamma_{2}\equiv \gamma_{2}\bar{\ell }\gamma_{1}$, such that each *γ_i _*is a telomere or an extremity of a marker from  G and *ℓ *is a substring composed of the markers which are between *γ*_1 _and *γ*_2 _in *A *(respectively in *B*) and contains no marker which also belongs to  G. The substring *ℓ *is the *label *of *v*. If *ℓ *is empty, the adjacency is said to be *clean*, otherwise it is said to be *labeled*. If a linear chromosome is composed only of markers which are not in  G, it is represented by an adjacency ○*ℓ*○.

#### DCJ operations

*A cut *performed on a genome *A *separates two adjacent markers of *A*. A cut affects a single adjacency *v *in *A*: it is done between two symbols of *v*, creating two open ends. A *double-cut and join *or *DCJ *applied on a genome *A *is the operation that performs cuts in two different adjacencies in *A*, creating four open ends, and joins these open ends in a different way. In other words, a DCJ rearranges two adjacencies in *A*, transforming them into two new adjacencies.

Consider a DCJ *ρ *applied to adjacencies *v*_1 _= *γ*_1_*ℓ*_1_*ℓ*_4_*γ*_4 _and *v*_2 _= *γ*_3_*ℓ*_3_*ℓ*_2_*γ*_2_, which creates *x*_1 _= *γ*_1_*ℓ*_1_*ℓ*_2_*γ*_2 _and *x*_2 _= *γ*_3_*ℓ*_3_*ℓ*_4_*γ*_4_. We represent such an operation as *ρ *= ({*γ*_1_*ℓ*_1_|*ℓ*_4_*γ*_4_, *γ*_3_*ℓ*_3_|*ℓ*_2_*γ*_2_} → {*γ*_1_*ℓ*_1_|*ℓ*_2_*γ*_2_, *γ*_3_*ℓ*_3_|*ℓ*_4_*γ*_4_}). The two adjacencies *v*_1 _and *v*_2 _are called the *sources*, while the two adjacencies *x*_1 _and *x*_2 _are called the *resultants *of *ρ *[8]. One or more labels among *ℓ*_1_, *ℓ*_2_, *ℓ*_3 _and *ℓ*_4 _can be equal to *ε *(the empty string), as well as one or more extremities among *γ*_1_, *γ*_2_, *γ*_3 _and *γ*_4 _can be equal to ○ (a telomere), A DCJ operation can correspond to several rearrangement events, such as an inversion, a translocation, a fusion or a fission [2].

#### Adjacency graph and the DCJ distance

**Figure 3:**
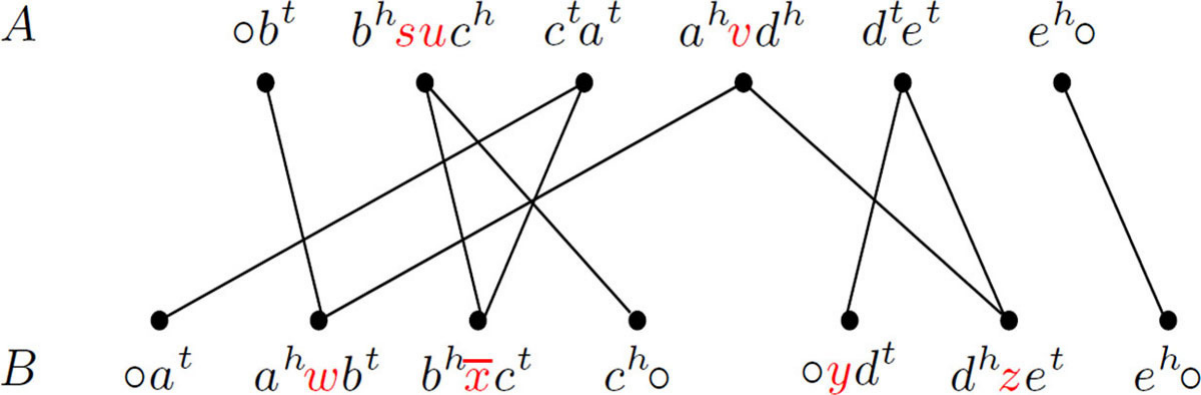
**For genomes *A *and *B*, the graph has one *BB *and two *AB*-paths**.

Given two genomes *A *and *B*, the *adjacency graph AG*(*A, B*) [3] is the bipartite multigraph whose vertices are the adjacencies of *A *and of *B *and that has one edge for each common extremity of a pair of vertices. The graph *AG*(*A, B*) is composed of connected components that alternate vertices in genome *A *and in genome *B*. Each component can be either a cycle, or an *AB-path *(which has one endpoint in genome *A *and the other in *B*), or an *AA-path *(which has both endpoints in genome *A*), or a *BB-path *(which has both endpoints in *B*). *A *special case of an *AA *or a *BB-*path is a *linear singleton*, that is a linear chromosome represented by an adjacency of type ○*ℓ*○. In Figure 3 we show the example of an adjacency graph.

Components with 3 or more vertices need to be reduced - by applying DCJ operations - to components with only 2 vertices, that can be cycles or *AB*-paths [8]. This procedure is called DCJ-sorting of *A *into *B*. The number of *AB*-paths in *AG*(*A, B*) is always even and a DCJ operation can be of three types [5]: it can either increase the number of cycles by one, or the number of *AB*-paths by two (*optimal *DCJ); or it does not affect the number of cycles and *AB*-paths (*neutral *DCJ); or it can either decrease the number of cycles by one, or the number of *AB*-paths by two (*counter-optimal *DCJ). We assign the same cost to any DCJ operation. For simplicity, we consider the DCJ cost equal to one. Then, the DCJ distance of *A *and *B*, denoted by *d_DCJ _*(*A, B*), corresponds to the minimum number of steps required to do a DCJ-sorting of *A *into *B *and is given by the following theorem.

**Theorem 1**([**3**]) *Given two genomes A and B without duplicated markers, we have *$d_{DCJ}\left(A,B\right)=\;|G|-\;c\;-\;\frac{b}{2}$ , *where * G* is the set of common markers and c and b are, respectively, the number of cycles and of AB-paths in AG *(*A, B*).

### The DCJ-indel model with distinct costs

Although the DCJ-model was defined in the previous sections for genomes with unequal contents, only the common markers were handled. In this section we explain how to deal with unique markers, that are markers which occur only in genome *A *and markers which occur only in genome *B*.

#### Indel operations

In order to deal with unique markers, we need operations that change the content of a genome. These operations can be an *insertion *or a *deletion *of a block of contiguous markers. Insertions and deletions can be jointly called *indel *operations. We consider a model in which an indel only affects the label of one single adjacency, by deleting or inserting contiguous markers in this label, with the restriction that an insertion cannot produce duplicated markers [5]. In other words, while sorting *A *into *B*, the indel operations are the steps in which the markers in  A are deleted and the markers in  B are inserted.

Given *ℓ*_3 _≠ *ε*, the deletion of *ℓ*_3 _from the adjacency *γ*_1_*ℓ*_1_*ℓ*_3_*ℓ*_2_*γ*_2 _is represented as (*γ*_1_*ℓ*_1_|*ℓ*_3_|*ℓ*_2_*γ*_2 _→ *γ*_1_*ℓ*_1_|*ℓ*_2_*γ*_2_), while the insertion of *ℓ*_3 _in the adjacency *γ*_1_*ℓ*_1_*ℓ*_2_*γ*_2 _is represented as (*γ*_1_*ℓ*_1_|*ℓ*_2_*γ*_2 _→ *γ*_1_*ℓ*_1_|*ℓ*_3_|*ℓ*_2_*γ*_2_). One or both extremities among *γ*_1 _and *γ*_2 _can be equal to ○ (a telomere), as well as one or both labels among *ℓ*_1 _and *ℓ*_2_, can be equal to *ε *(the empty string). Observe that at most one chromosome can be entirely deleted or inserted at once. Moreover, since duplications are not allowed, an insertion of a marker that already exists is not allowed. Consequently, in this model, it is not possible to apply insertions and/or deletions involving the markers in  G.

Given two genomes *A *and *B*, the *DCJ-indel distance *of *A *and *B*, denoted by $d_{DCJ}^{id}\left(A,B\right)$, is the minimum cost of a DCJ-indel sequence of operations which sorts *A *into *B*, assigning the cost of 1 to each DCJ and a positive cost *w *≤ 1 to each indel operation. If *w *= 1, the DCJ-indel distance corresponds exactly to the minimum number of steps required to sort *A *into *B *[5].

#### Runs, indel-potential and the DCJ-indel distance

**Figure 4:**
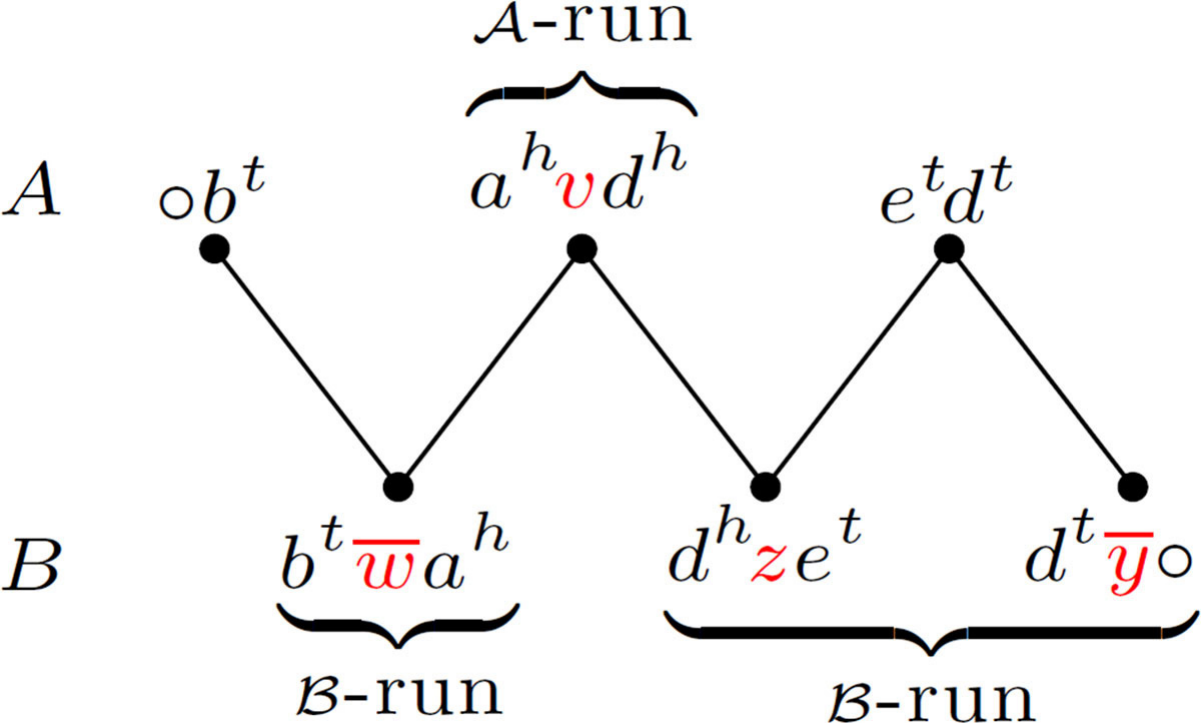
**An *AB*-path with 3 runs (extracted from Figure 3)**. The first and the second runs are compact, while the third run is long and composed of three vertices.

Let us recall the concept of *run*, introduced by Braga *et al. *[5]. Given two genomes *A *and *B *and a component *C *of *AG*(*A, B*), a *run *is a maximal subpath of *C*, in which the first and the last vertices are labeled and all labeled vertices belong to the same genome (or partition). A run is then a subpath of a component and can be represented by its list of vertices. A vertex *v *that corresponds to an entire run is called a *compact*-*run*. If a run is not compact, it is a long-run. An example of a component with 3 runs is given in Figure 4. A run in genome *A *is also called an  A-run, and a run in genome *B *is called a  B-run. We denote by Λ(*C*) the number of runs in a component *C*. While a path can have 0 or any positive number or runs, a cycle has either 0, 1, or an even number of runs.

A set of labels of one genome can be accumulated with DCJs. In particular, when we apply optimal DCJs on only one component of the adjacency graph, we can accumulate an entire run into a single adjacency [5]. It is possible to do a *separate DCJ*-*sorting *using only optimal DCJs in any component *C *of *AG*(*A, B*) [8]. We denote by *d_DCJ _*(*C*) the number of optimal DCJ operations used for DCJ-sorting *C *separately (*d_DCJ _*(*C*) depends only on the number of vertices or, equivalently, the number of edges of *C *[8]). The DCJ distance can also be re-written as *d_DCJ_*(*A, B*) = ∑_*c*∈*AG*(*A, B*) _*d_DCJ_*(*C*).

**Figure 5:**
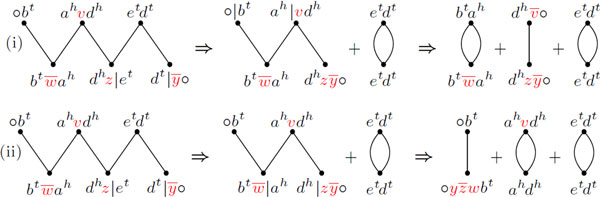
**Two optimal sequences for DCJ-sorting an *AB*-path with Λ = 3 (the cuts of each DCJ in each sequence are represented by "|")**. In (i) the overall number of runs in the resulting components is three, while in (ii) the resulting components have only two runs. Indeed, in this case, the best we can have is the indel-potential λ = 2.

Runs can be merged by DCJ operations. Consequently, during the optimal DCJ-sorting of a component *C*, we can reduce its number of runs. The *indel-potential *of *C*, denoted by *λ*(*C*), is defined by Braga *et al. *[5] as the minimum number of runs that we can obtain doing a separate DCJ-sorting in *C *with optimal DCJ operations. An example is given in Figure 5.

The indel-potential of a component depends only on its number of runs:

**Proposition 1 **([5]) *Given two genomes A and B and a component C of AG*(*A, B*), *the indel-potential of C is given by *$\lambda \left(C\right)=\left\lceil \frac{\Lambda \left(C\right)+1}{2}\right\rceil$, *if *Λ(*C*) ≥ 1. *Otherwise, if *Λ(*C*) = 0, *then λ*(*C*) = 0.

Let *λ*_0 _and *λ*_1 _be, respectively, the sum of the indel-potentials for the components of the adjacency graph before and after a DCJ operation *ρ*, and let Δ*λ*(*ρ*) = *λ*_1 _- *λ*_0_. If *ρ *is an optimal DCJ acting on two adjacencies of a single component of the graph, the definition of indel-potential implies Δ*λ*(*ρ*) ≥ 0. We also know that Δ*λ*(*ρ*) ≥ 0, if ρ is counter-optimal, and Δ*λ*(*ρ*) ≥ -1, if *ρ *is neutral [5]. This allows us to exactly compute $d_{DCJ}^{id}\left(C\right)$, that is the DCJ-indel distance of a component *C *of $AG\left(A,B\right):d_{DCJ}^{id}\left(C\right)=d_{DCJ}\left(C\right)+w\lambda \left(C\right)$[6]. We can then derive the following upper bound for the DCJ-indel distance:

**Lemma 1 **([6]) *Given two genomes A and B without duplicated markers and a positive indel cost w ≤ *1, *we have*

$$d_{DCJ}^{id}\left(A,B\right)\leq d_{DCJ}\left(A,B\right)+w\underset{C\in AG\left(A,B\right)}{\sum }\lambda \left(C\right).$$

##### Recombinations

**Figure 6:**
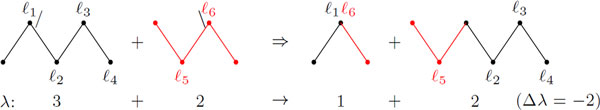
**This recombination is a neutral DCJ that has Δ*λ *= -2 (we represent only the labels of the adjacencies, the cuts of the recombination are represented by "/"and "\")**.

Until this point, we have explored the possible effects of any DCJ that is applied to two adjacencies belonging to a single component of the graph. However, there is another type of DCJ that must be considered. A DCJ operation *ρ *applied to adjacencies belonging to two different components is called a *recombination *and can have Δ*λ*(*ρ*) < 0 [5]. Thus, depending on the value of *w *and on whether the recombination is an optimal, a neutral or a counter-optimal DCJ, a recombination with Δ*λ*(*ρ*) < 0 can lead to a sorting sequence with lower cost. As an example, a neutral recombination with Δ*λ *= -2 is represented in Figure 6.

Although many different recombinations can occur, it is possible to explore the space of recombinations in linear time and compute the maximum deduction that we can obtain with respect to the upper bound of Lemma 1 [6].

## Results

In this section we develop a restricted DCJ-indel sorting algorithm, from which we can derive an upper bound for the restricted DCJ-indel distance.

### Chained operations

Let us generalize to the DCJ-indel model a concept introduced in [8]. Let *s *= *ρ*_1_*ρ*_2 _... *ρ*_*n*-1_*ρ_n _*be a DCJ-indel sequence of operations sorting genome *A *into genome *B*. Two consecutive operations *ρ*_*i *_and *ρ*_*i*+1 _of *s *are said to be independent when no source of *ρ*_*i*+1 _is a resultant of *ρ_i_*. Otherwise, *ρ*_*i*+1 _use as a source a resultant from *ρ_i_*. In this case, the operations *ρ_i _*and *ρ*_*i*+1 _are said to be *chained*.

### Bi-directional approach

Although in general a sorting algorithm is conceived to follow a single direction, in which all operations are applied on the initial genome, here we design a bi-directional algorithm, in which some operations are applied on genome *A *and the others are applied on genome *B*. Running a bi-directional algorithm we actually transform genomes *A *and *B *into an intermediate genome *I*. However, with the operations that transform *A *and *B *into *I*, we can derive an optimal sequence of operations simply sorting genome *A *into *B*. Given any DCJ or indel operation *ρ *= (*X *→ *Y*), the *inverse *of *ρ *is *ρ*^-1 ^= (*Y *→ *X*) [5]. This notation can also be extended to a sequence of operations: given a sequence *s *= *ρ*_1_*ρ*_2 _... *ρ_n _*, we have $s^{-1}=\rho_{n}^{-1}\rho_{n-1}^{-1}\cdots \rho_{2}^{-1}\rho_{1}^{-1}$. Observe that the inverse of a deletion is an insertion, and *vice*-*versa*.

**Proposition 2 **([5]) *Given two genomes A and B, and a pair of sequences s*_1 _*and s*_2 _*composed of DCJ and indel operations applied respectively on genomes A and B, transforming both A and B into an intermediate genome I, such that *$|s_{1}|+|s_{2}|=d_{DCJ}^{id}\left(A,B\right)$, *then *$s_{1}s_{2}^{-1}$*is an optimal sequence of DCJ and indel operations that transforms A into B*.

**Figure 7:**
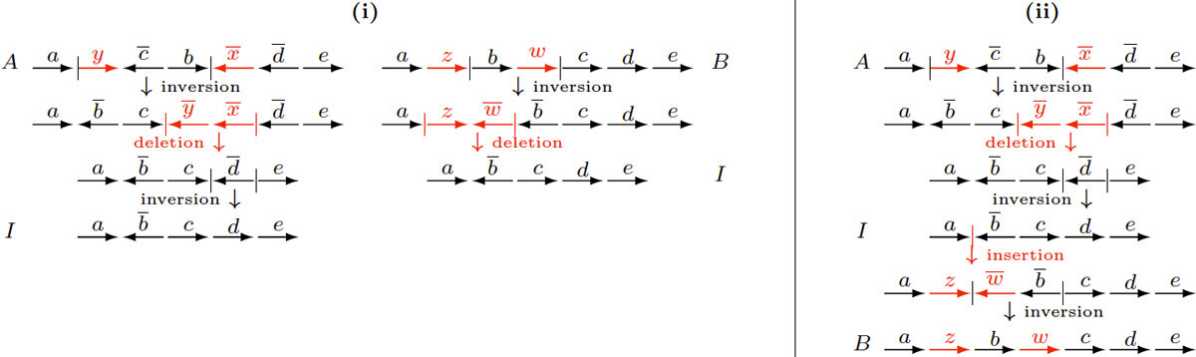
**(i) Two sequences of lengths 3 and 2, sorting $A=\left\{\circ ay\bar{c}b\bar{x}\bar{d}e\circ \right\}$ and *B *= {○*azbwcde*○} into $I=\left\{\circ a\bar{b}cde\circ \right\}$**. (ii) A corresponding sequence of length 5 sorting A into B. (Unique markers are represented in red.).

Figure 7 illustrates the generation of a sequence of operations sorting *A *into *B *from a bi-directional sequence of operations.

#### Accumulating x splitting labels

A DCJ that accumulates labels is always applied to two labeled adjacencies and results into a clean adjacency and an adjacency containing the concatenation of the labels of the original adjacencies. In general, we can represent such an accumulating DCJ *ρ *by ({*γ*_1_*ℓ*_1_|*γ*_4_, *γ*_3_|*ℓ*_2_*γ*_2_}→{*γ*_1_*ℓ*_1_|*ℓ*_2_*γ*_2_, *γ*_3_|*γ*_4_}). If *ρ *accumulate labels of an  A-run, it is denoted by $A_{A}^{A}≻B$. Similarly, if *ρ *accumulates labels of a  B-run, it is denoted by $B_{B}^{B}≻A$.

The inverse of an accumulating DCJ *ρ *is a splitting DCJ *ρ*^-1 ^= ({*γ*_1_*ℓ*_1_|*ℓ*_2_*γ*_2_, *γ*_3_|*γ*_4_} → {*γ*_1_*ℓ*_1_|*γ*_4_, *γ*_3_|*ℓ*_2_*γ*_2_}). Observe that, if *ρ *is applied on *A, ρ*^-1 ^is applied on *B *and split a label of an  A-run. In other words, the inverse of an $A_{A}^{A}≻B$ is a DCJ applied on *B *that separates vertices belonging to the same  A-run in two different cycles, denoted by $B{≺}_{A}^{A}A$. Similarly, the inverse of a $B_{B}^{B}≻A$ is a DCJ applied on A that separates vertices belonging to the same  B-run, denoted by $A{≺}_{B}^{B}B$. An $A{≺}_{B}^{B}B$ or a $B{≺}_{A}^{A}A$ is called an *inverted*-*split*. In Table 1 we summarize the operations described above.

**Table 1:** Accumulating and splitting DCJ operations

Operation	Direction	Effect	Inverse
$A_{A}^{A}≻B$	*A*→*B *	Accumulate labels of an A-run	${\left(A_{A}^{A}≻B\right)}^{-1}=B{≺}_{A}^{A}A$

$A{≺}_{B}^{B}B$	*A*→*B *	Inversely split label of a B-run	${\left(A{≺}_{B}^{B}B\right)}^{-1}=B_{B}^{B}≻A$

$B_{B}^{B}≻A$	*B*→*A *	Accumulate labels of a B-run	${\left(B_{B}^{B}≻A\right)}^{-1}=A{≺}_{B}^{B}B$

$B{≺}_{A}^{A}A$	*B*→*A *	Inversely split label of an A-run	${\left(B{≺}_{A}^{A}A\right)}^{-1}=A_{A}^{A}≻B$

#### Accumulation-deletion x insertion-split

Let *n *be a positive integer, such that *n *≥ 2 and let *r*_1 _= *v*_1_*x*_1_*v*_2_*x*_2 _... *v_i_x_i _*... *v_j_x_j _*... *v*_*n*-1_*x*_*n*-1_*v_n _*be a long-run, in which *v*_1 _and *v*_*n *_are labeled, each *v_k _*(2 ≤ *k *≤ *n *- 1) can also be labeled and all *x_k _*(1 ≤ *k *≤ *n *- 1) are clean. We say that two vertices *v*_*i *_and *v*_*j *_(1 ≤ *i *<*j *≤ *n*) in *r*_1 _are *partners *if *v*_*i *_and *v*_*j *_are labeled and all vertices between *v*_*i *_and *v*_*j *_in *r*_1 _are clean. We can apply an accumulating DCJ on the two partners *v*_*i *_and *v_j_*, accumulating their labels into a new vertex *v*_*i*-*j*_, reducing *r*_1 _to *r*_2 _= *v*_1_*x*_1_*v*_2_*x*_2 _... *v*_*i*-1_*x*_*i*-1_*v*_*i*-*j*_*x*_*j*_*v*_*j*+1_*x*_*j*+1 _... *v*_*n*-1_*x*_*n*-1_*v_n_*. The subsequent step of accumulation then occurs between two partners of *r*_2_, reducing *r*_2 _to *r*_3_, and so on. Assuming that the initial *r*_1 _has *m *≤ *n *labeled vertices, we need to apply *m *- 1 accumulating operations. In the end of the process, we obtain the compact-run *r_m_*, that corresponds to a single vertex whose label is the accumulation of all labels of *r*_1_. Observe that all labeled vertices will be used in some accumulating DCJ, until the compact-run *r_m _*is obtained.

**Figure 8:**
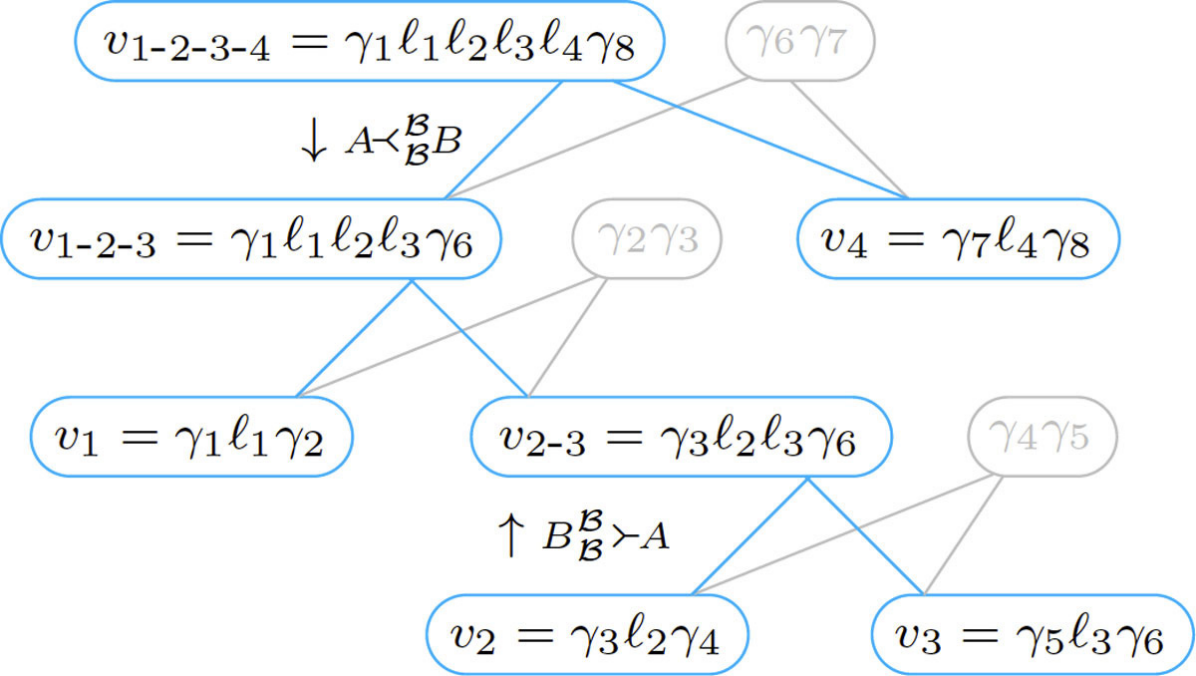
**The rooted tree of an accumulation of a  B-run is built from the leafs to the root (bottom to up)**. Inversely, the rooted tree of an inverted-split of a  B-run is built from the root to the leafs (top to down).

As an example, take *v*_1 _= *γ*_1_*ℓ*_1_*γ*_2_, *x*_1 _= *γ*_2_*γ*_3_, *v*_2 _= *γ*_3_*ℓ*_2_*γ*_4_, *x*_2 _= *γ*_4_*γ*_5_, *v*_3 _= *γ*_5_*ℓ*_3_*γ*_6_, *x*_3 _= *γ*_6_*γ*_7_, *v*_4 _= *γ*_7_*ℓ*_4_*γ*_8_, with all *ℓ_k _*≠ *ε *and let *r*_1 _= *v*_1_*x*_1_*v*_2_*x*_2_*v*_3_*x*_3_*v*_4 _be a  B-run. We can start the accumulation with a DCJ of type $B_{B}^{B}≻A$ on partners *v*_2 _and *v*_3_, creating *v*_2-3 _= *γ*_3_*ℓ*_2_*ℓ*_3_*γ*_6 _and *γ*_4_*γ*_5_, reducing *r*_1 _to *r*_2 _= *v*_1_*x*_1_*v*_2-3_*x*_3_*v*_4_. We then apply another DCJ of type $B_{B}^{B}≻A$ on partners *v*_1 _and *v*_2-3_, creating *v*_1-2-3 _= *γ*_1_*ℓ*_1_*ℓ*_2_*ℓ*_3_*γ*_6 _and *γ*_2_*γ*_3_, reducing *r*_2 _to *r*_3 _= *v*_1-2-3_*x*_3_*v*_4_. Finally, we apply a DCJ of type $B_{B}^{B}≻A$ on partners *v*_1-2-3 _and *v*_4_, creating *v*_1-2-3-4 _= *γ*_1_*ℓ*_1_*ℓ*_2_*ℓ*_3_*ℓ*_4_*γ*_8 _and *γ*_6_*γ*_7_, reducing *r*_3 _to *r*_4 _= *v*_1-2-3-4_. If we follow the accumulation of a run, considering only the labeled vertices, we obtain a rooted tree that is built from the leafs to the root (see Figure 8). The root of the tree indicates the possible positions of a deletion.

The inversion of the run accumulation described in the example above is the inverted-split of the label of the compact-run *r*_4 _= *v*_1-2-3-4 _into the labeled vertices *v*_1 _= *γ*_1_*ℓ*_1_*γ*_2_, *v*_2 _=*γ*_3_*ℓ*_2_*γ*_4_, *v*_3 _= *γ*_5_*ℓ*_3_*γ*_6 _and *v*_4 _= *γ*_7_*ℓ*_4_*γ*_8_. We start by applying a $B{≺}_{A}^{A}A$ DCJ on *v*_1-2-3-4 _= *γ*_1_*ℓ*_1_*ℓ*_2_*ℓ*_3_*ℓ*_4_*γ*_8 _and *γ*_6 _*γ*_7_, obtaining *v*_1-2-3 _= *γ*_1_*ℓ*_1_*ℓ*_2_*ℓ*_3_*γ*_6 _and *v*_4 _= *γ*_7_*ℓ*_4_*γ*_8_. We then apply a $B{≺}_{A}^{A}A$ on *v*_1-2-3 _and *γ*_2_*γ*_3_, obtaining *v*_2 _= *γ*_1_*ℓ*_1_*γ*_1 _and *v*_2-3 _= *γ*_3_*ℓ*_2_*ℓ*_3_*γ*_6_. Finally we apply a $B{≺}_{A}^{A}A$ on *v*_2-3 _and *γ*_4_*γ*_5_, obtaining *v*_2 _= *γ*_3_*ℓ*_2_*γ*_4 _and *v*_3 _=*γ*_5_*ℓ*_3_*γ*_6_. If we follow the inverted-split of a run, considering only the labeled vertices, we obtain a rooted tree that is built from the root to the leafs (see Figure 8 again). In this case, the first inverted-split defines the root. Then, each one of the subsequent inverted-splits must be chained with a DCJ in this tree. The root of the tree indicates the possible positions of an insertion.

### An indel does not have to occur while a circular chromosome exists

We now show that an indel must not be applied while a circular chromosome exists.

Proposition 3 shows that an insertion can always be "moved up" in a DCJ-indel sorting sequence.

**Proposition 3 ***Let s *= *ρ*_1_*ρ*_2 _... *ρ*_*n*-1_*ρ_n _be a DCJ-indel sequence sorting genome A into genome B, such that, for an integer *1 ≤ *i *<*n, ρ_i _is a DCJ operation and ρ*_*i*+1 _*is an insertion. Then ρ_i _ρ*_*i*+1 _*can be replaced θ*_1_*θ*_2_, *such that θ*_1 _*is an insertion and θ*_2 _*is a DCJ and s' *= *ρ*_1_*ρ*_2 _... *ρ*_*i*-1_*θ*_1_*θ*_2_*ρ*_*i*+ 2 _... *ρ*_*n*-1_*ρ_n _is also a DCJ-indel sequence sorting genome A into genome B*.

*Proof: *Observe that, if *ρ*_*i *_and *ρ_i_*+1 are independent, it is easy to see that they can be simply switched, that is: *θ*_1 _= *ρ*_*i*+1 _and *θ*_2 _= *ρ_i_*. We still need to examine the case in which *ρ_i _*and *ρ*_*i*+1 _are chained.

Observe that a DCJ in any optimal sorting scenario either accumulates or does not change the composition of runs. Take *ρ*_*i *_= ({*γ*_1_*ℓ*_1_|*γ*_4_, *γ*_3_|*ℓ*_2_*γ*_2_} → {*γ*_1_*ℓ*_1_|*ℓ*_2_*γ*_2_, *γ*_3_|*γ*_4_}). Furthermore, since an insertion in any optimal sequence is performed without breaking any existing label, without loss of generality, take *ρ_i+1_* = (*γ*_1_*ℓ*_1_*ℓ*_2_|*γ*_2 _→ *γ*_1_*ℓ*_1_*ℓ*_2_|*ℓ*_3_|*γ*_2_). Then *ρ*_*i*_*ρ*_*i*+1 _could be replaced by: *θ*_1 _= (*γ*_3_*ℓ*_2_|*γ*_2 _→ *γ*_3_*ℓ*_2_|*ℓ*_3_|*γ*_2_) followed by *θ*_2 _= ({*γ*_1_*ℓ*_1_|*γ*_4_, *γ*_3_|*ℓ*_2_*ℓ*_3_*γ*_2_} → {*γ*_1_*ℓ*_1_|*ℓ*_2_*ℓ*_3_*γ*_2_, *γ*_3_|*γ*_4_}).

Similarly, a deletion can always be "moved down" in a DCJ-indel sorting sequence.

**Proposition 4 ***Let s *= *ρ*_1_*ρ*_2 _... *ρ*_*n*-1_*ρ*_*n *_*be a DCJ-indel sequence sorting genome A into genome B, such that, for an integer *1 ≤ *i *<*n, ρ_i _is a deletion and ρ*_*i*+1 _*is a DCJ operation. Then ρ_i_ρ*_*i*+1 _*can be replaced by θ*_1_*θ*_2_, *such that θ*_1 _*is a DCJ operation and θ*_2 _*is a deletion and s' *= *ρ*_1_*ρ*_2 _... *ρ*_*i*-1_*θ*_1_*θ*_2_*ρ*_*i*+2_ ... *ρ*_*n*-1_*ρ_n _is also a DCJ-indel sequence sorting genome A into genome B*.

*Proof: *Analogous to the proof of Proposition 3.    □

From the previous propositions we observe that finding a position to perform an indel imposes no difficulties to design a restricted DCJ-indel sorting algorithm. The trick is how to determine the DCJ part of the sorting sequence, so that we reincorporate each circular chromosome after its creation and achieve the indel-potential per component.

### Restricted DCJ-indel sorting

Basically, our approach disregards recombinations and sorts the components of the graph separately, using optimal DCJ operations to achieve the minimum number of indels per component, that is given by the indel-potential. In this way, we achieve the distance given by the upper bound of Lemma 1, as we will see in the remainder of this section.

#### Capping

Disregarding recombinations, we can first perform the genome capping, a technique that helps us to avoid difficulties and special cases produced by telomeres: we adjoin new markers (caps) to the ends of the chromosomes (and new chromosomes composed of caps only, if necessary) so that we do not change the distance and we do not have to worry about telomeres [4]. After the capping, the two genomes have the same number of chromosomes and the corresponding adjacency graph contains only clean paths of size 1 and cycles. Recall that, since *AG*(*A, B*) is bipartite, all cycles have even length and can have 0, 1 or an even number of runs. Capped genomes can be then sorted with translocations (which mimic also fusions and fissions), inversions, circular chromosome excisions and reincorporations.

#### Merging runs in cycles

An important step of the DCJ-indel sorting is to merge runs in cycles with at least 4 runs, so that the indel-potential for each cycle is achieved.

**Proposition 5 ***The indel-potential of a cycle C with at least 4 vertices and 2 or more runs can be achieved by extracting from C a cycle with a single run*.

*Proof: *For any positive integer i let $\lambda \left(i\right)=\left\lceil \frac{i+1}{2}\right\rceil$. If Λ(*C*) = 2, we can split *C *into two cycles containing a single run each, and the indel-potential is preserved. For any cycle *C *with 4 or more runs, since the number of runs in this case is always even, we have $\lambda \left(i\right)=\frac{i}{2}+1$. We then denote by λ' the alternative potential, obtained by extracting cycles with a single run from *C*. Observe that, for any *i *= 4, 6, 8, ..., *λ*'(*i*) = *λ*(*i *- 2) + 1. It is easy to check the base case, that is $\lambda^{\prime }\left(4\right)=\lambda \left(2\right)+1=2+1=3=\frac{4}{2}+1=\lambda \left(4\right)$. By induction, for *i *= 6, 8, 10 ..., we have $\lambda^{\prime }\left(i\right)=\lambda \left(i-2\right)+1=\frac{i-2}{2}+1+1=\frac{i}{2}+1=\lambda \left(i\right)$    □

#### Chromosome reincorporation

In the restricted sorting of linear genomes a circular chromosome has to be immediately reincorporated after its excision - these two consecutive operations mimic either a transposition or a block-interchange [2,4]. As we have seen before, the general DCJ-indel sorting is bi-directional - the operations can be applied on genome A or B, depending on whether we accumulate runs in A or in B. However, when a DCJ creates a circular chromosome, we need to apply the subsequent DCJ on the same genome, and it is not easy to see how this interferes with the indel-potential of *AG*(*A, B*).

Suppose that a DCJ performed an excision of a circular chromosome. Let (*v*_1_, *v*_2_) be a pair of vertices, such that *v*_1 _and *v*_2 _are in the same genome and belong to the same cycle in *AG*(*A, B*), *v*_1 _is an adjacency at the circular chromosome and *v*_2 _is an adjacency at a linear chromosome. The pair (*v*_1_, *v*_2_) is called a link. Since *v*_1 _and *v*_2 _are in the same cycle, a chromosome reincorporation can always be done by applying a DCJ on the two vertices *v*_1 _and *v*_2 _[8].

The cycle to which a link (*v*_1_, *v*_2_) belongs is called a *connection cycle*. Let *C *be a connection cycle of *AG*(*A, B*) with 2*k *≥ 4 vertices. Since *C *has *k *vertices in each genome, there are at least *k *- 1 and at most $\left\lceil \frac{k}{2}\right\rceil .\left\lfloor \frac{k}{2}\right\rfloor$ distinct links in *C*.

The two vertices *v*_1 _and *v*_2 _of a link in a connection cycle *C *are connected by two distinct subpaths of *C*. The distance between *v*_1 _and *v*_2 _is given by the number of edges in the shortest path connecting them. Since both *v*_1 _and *v*_2 _are in the same genome, this distance is always even and positive. If the distance between *v*_1 _and *v*_2 _is 2, *v*_1 _and *v*_2 _have a common neighbor, and (*v*_1_, *v*_2_) is called a *short-link*.

**Proposition 6 ***After the excision of a circular chromosome by a DCJ, there is at least one short-link in AG*(*A, B*).

*Proof: *Suppose that the circular chromosome is in genome *A*. If *AG*(*A, B*) contained no connection cycle, genome B would also have a circular chromosome, which would be a contradiction. Let *C *= *v*_1_*x*_1_*v*_2_*x*_2 _... *v*_*n*_*x*_*n *_be a connection cycle in *AG*(*A, B*), in which the vertices *v*_1_, ..., *v*_*n *_are in *A *and the vertices *x*_1_, ..., *x*_*n *_are in *B*, and let (*v_i_, v_j_*) be a link in *C *such that *v*_*i *_is in the circular chromosome and *v*_*j *_is in a linear chromosome of *A*. Consider without loss of generality that *i *<*j*. Then take the vertex *v_k_, i *≤ *k *<*j*, such that *k *is the largest index of a vertex between *v*_*i *_and *v*_*j *_belonging to the circular chromosome. Then (*v_k_, v*_*k*+1_) is a short-link.    □

In order to find out whether the indel-potential of the connection cycle *C *can be preserved after applying a DCJ on a certain link (*v*_1_, *v*_2_), basically we need to analyze how the connection cycle *C *is split, by analyzing the vertices that are between *v*_1 _and *v*_2 _in *C*.

We focus on the short-links only. Let (*v*_1_,*v*_2_) be a short-link in a connection cycle *C*, such that *v*_1 _= *γ*_1_*ℓ*_1_*γ*_2 _and *v*_2 _= *γ*_3_*ℓ*_2_*γ*_4 _(*ℓ*_1 _and *ℓ*_2 _can be equal to *ε*). Without loss of generality, let *z *= *γ*_2_*ℓ*_3_*γ*_3 _be the common neighbor of *v*_1 _and *v*_2 _(*ℓ*_3 _can also be equal to *ε*). We then define the optimal DCJ *ρ*(*v*_1_, *v*_2_) = ({*v*_1_, *v*_2_} → {*x*_1_, *x*_2_}), such that *x*_1 _= *γ*_2_*γ*_3 _and *x*_2 _= *γ*_1_*ℓ*_1_*ℓ*_2_*γ*_4_. Observe that *ρ*(*v*_1_, *v*_2_) always extracts *z *together with a new clean vertex *x*_1 _into a cycle, and accumulates the labels of *v*_1 _and *v*_2 _into a new vertex *x*_2_, which is extracted into a cycle with the remaining vertices of *C*. There are three different cases:

1. *Gaps: *If the two vertices of a short-link have a clean common neighbor, it is called a *gap*. A DCJ applied to a gap of a connection cycle *C *splits *C *into a clean cycle *C' *and a cycle *C'' *that has the same indel-potential of *C*.

2. *Compact-runs: *Let (*v*_1_, *v*_2_) be a short-link in *AG*(*A, B*), such that the common neighbor *z *of *v*_1 _and *v*_2 _is a compact-run. An optimal DCJ *ρ*(*v*_1_, *v*_2_) extracts the compact-run *z *and a new clean vertex into a new cycle. According to Proposition 5, *ρ*(*v*_1_, *v*_2_) preserves the indel-potential of *AG*(*A, B*).

3. *Inverted-splits: *If a short-link (*v*_1_, *v*_2_) is not a gap nor is separated by a compact-run, only one possiblity remains: the common neighbor *z *of *v*_1 _and *v*_2 _is labeled and belongs to a long-run *r*. Observe that an optimal DCJ *ρ*(*v*_1_, *v*_2_) splits *C *into a cycle *C' *containing a new clean vertex and *z *(Λ(*C'*) = 1) and a cycle *C*'' containing all remaining runs of *C *and the remaining vertices of *r*, that is, we have Λ(*C*'') = Λ(*C*).

Although the overall indel-potential seems to be increased, the DCJ described above is an inverted-split of type $A{≺}_{B}^{B}B$ if the circular chromosome is in *A *and *r *is in *B *(or, symmetrically, of type $B{≺}_{A}^{A}A$, if the circular chromosome is in *B *and *r *is in *A*). We have seen that inverted-splits, if properly applied, do the backtracing of the insertion position of a run in the opposite genome and do not increase the indel-potential of *AG*(*A, B*).

It is important to guarantee that, after applying a DCJ that inversely splits a run *r*_1 _and another DCJ that inversely splits another run *r*_2_, the runs *r*_1 _and *r*_2 _are not merged. We do this by simply extracting the residual part of an inversely split run into a new cycle. Furthermore, during the merging or accumulation of runs, a run *r *can be inversely split by successive DCJs. In this case, we need to guarantee that each new inverted-split of *r *is either the first or chained with one of the previous inverted-splits.

We can always reincorporate the circular chromosome with a DCJ applied to any short-link (*v*_1_, *v*_2_), except if *ρ*(*v*_1_, *v*_2_) splits a run *r *that is already inversely split and *ρ*(*v*_1_, *v*_2_) cannot be chained with a previous inverted-split of *r*. However, in this case, *r *will be separated alone in a cycle (each run is immediately separated after its first inverted-split).

After an excision, suppose that the circular chromosome is in genome *A *(respectively in *B*). Let *C *be a connection cycle in *AG*(*A, B*). For each vertex *v *of *C *in *A *(respectively in *B*), there is at least one link containing *v*. Due to this fact, when we have a cycle containing a single inversely split run *r*, it is easy to find a link chained with a previous inverted split of *r*.

**Proposition 7 ***If a connection cycle C with a single run r has links in one genome and its run r is in the other genome, we can always reincorporate the circular chromosome and preserve the indel-potential*.

*Proof: *Let *C *have links in genome *A*. Each short-link of *C *is either a gap, or a compact-run, or the first inverted-split of the  B-run *r*. Otherwise, *C *has in genome *A *a vertex *v *that was created by a previous inverted-split *ρ *of *r*. Since each vertex of *C *in *A *is part of a link, we can choose a link that contains *v *and, consequently, is chained with *ρ*.    □

#### The sorting algorithm and an upper bound for the restricted DCJ-indel distance

We put everything together in Algorithm 1 (Additional file 1) and describe the sorting of capped genomes for the restricted model, in which each circular chromosome is reincorporated immediately after its creation. Applying this procedure we can find a sequence of optimal DCJs that sort *A *into *B *while preserving the indel-potential. In other words, this algorithm results in a sorting sequence in the restricted model that has exactly the same cost given by the upper bound of Lemma 1.

## Conclusions

In this work we have presented a method to compute a restricted DCJ-indel sequence of operations that sort a linear genome into another linear genome. This method leads to a tight upper bound for the restricted DCJ-indel distance. The general DCJ-indel distance can be computed exactly and is a lower bound for the restricted DCJ-indel distance. However, the question whether these bounds are equal, meaning that both distances are equal, remains open.

## Competing interests

The authors declare that they have no competing interests.

## Authors' contributions

PHS, MDVB, RM and SD have elaborated the model, proved the results and written the paper.

## 

**Algorithm 1 **Restricted sorting of genome A into B with optimal DCJs and indels

**Input: **Two linear genomes *A *and *B*

**Output: **A restricted sequence of DCJ and indel operations sorting *A *into *B*

     cap genomes *A *and *B*;

        [MERGING:]

     *r *← *null*;

     **if **there is a cycle *C *∈ *AG*(*A*,*B*) with at least 4 vertices and at least 2 runs **then**

         *r *← run from *C*;

     **while ***r *≠ *null ***do**

         extract *r *into a cycle; [*this preserves the indel-potential of AG*(*A*,*B*) *according to Proposition *5]

         *r *← *null*;

         **if **a circular chromosome was created **then**

             find a short-link (*v*_1_, *v*_2_); [*Proposition 6*]

             **if **(*v*_1_, *v*_2_) is a gap or a compact-run **then**

                   apply the optimal DCJ *ρ*(*v*_1_, *v*_2_);

             **else**

                let *r*_1 _be the run that would be inversely split by *ρ*(*v*_1_, *v*_2_);

                **if ***ρ*(*v*_1_, *v*_2_) is the first inverted-split of *r*_1 _**then**

                      apply the optimal DCJ *ρ*(*v*_1_, *v*_2_);

                    let *r*_2 _be the residual part of *r*_1_;

                    **if ***r*_2 _is in a cycle with more runs **then**

                       *r *← *r*_2_; [*extract r_2 _from its cycle in the next step*]

                  **else**

                       [*r*_1 _*was inversely split before and is separated alone in cycle*]

                    find a link (*x*_1_, *x*_2_) such that *x*_1 _is a vertex created by a previous inverted-split of *r*_1_; *Proposition *7]

                      apply the optimal DCJ *ρ*(*x*_1_, *x*_2_);

         **if ***r *= *null *and there is a cycle *C *∈ *AG*(*A*,*B*) with at least 4 vertices and at least 2 runs **then**

             *r *← run from *C*;

        [ACCUMULATING: *(each cycle with 4 or more vertices has at most one run)*]

     **while **there is a long-run r in *AG*(*A*,*B*) **do**

            apply an optimal DCJ accumulating the labels of two partners of *r*;

         **if **a circular chromosome was created **then**

             find a short-link (*v*_1_, *v*_2_); [*Proposition 6*]

             **if **(*v*_1_, *v*_2_) is a gap or a compact-run **then**

            apply the optimal DCJ *ρ*(*v*_1_, *v*_2_);

             **else**

             let *r*_1 _be the run that would be inversely split by *ρ*(*v*_1_, *v*_2_);

             **if ***ρ*(*v*_1_, *v*_2_) is the first inverted-split of *r*_1 _**then**

                apply the optimal DCJ *ρ*(*v*_1_, *v*_2_);

             **else**

                [*r*_1 _*was inversely split before and is separated alone in cycle*]

             find a link (*x*_1_, *x*_2_) such that x_1 _is a vertex created by a previous inverted-split of *r*_1_; [*Proposition *7]

             apply the optimal DCJ *ρ*(*x*_1_, *x*_2_);

        [DCJ-SORTING: *(each remaining cycle with 4 or more vertices has at most one compact-run)*]

     **while **there is cycle *C *∈ *AG*(*A*,*B*) with at least 4 vertices **do**

         extract a cycle from *C*, with an optimal DCJ applied on genome *A*;

         **if **a circular chromosome was created **then**

            find a short-link (*v*_1_, *v*_2_); [*Proposition 6*]

            [*at this stage this short-link is a gap or a compact-run*]

            apply the optimal DCJ *ρ*(*v*_1_, *v*_2_);

invert all DCJs applied on genome *B*;

insert each  B-run *r *before the first inverted-split of *r*;

move up insertions that occur in circular chromosomes;

delete all  A-runs from the DCJ-sorted components;

## Supplementary Material

Additional file 1 Click here for file

## References

[B1] Hannenhalli S Pevzner P Transforming men into mice (polynomial algorithm for genomic distance problem) Proc of FOCS 1995 1995 581 592

[B2] Yancopoulos S Attie O Friedberg R Efficient sorting of genomic permutations by translocation, inversion and block interchange Bioinformatics 2005 21 3340 3346 10.1093/bioinformatics/bti535 15951307

[B3] Bergeron A Mixtacki J Stoye J A unifying view of genome rearrangements Proc of WABI 2006 LNBI 2006 4175 163 173

[B4] Kovác J Warren R Braga MDV Stoye J Restricted DCJ Model: rearrangement problems with chromosome reincorporation Journal of Computational Biology 2011 18 9 1231 1241 10.1089/cmb.2011.0116 21899428

[B5] Braga MDV Willing E Stoye J Double Cut and Join with Insertions and Deletions Journal of Computational Biology 2011 18 9 1167 1184 10.1089/cmb.2011.0118 21899423

[B6] da Silva PH Braga MDV Machado R Dantas S DCJ-indel distance with distinct operation costs Proceedings of WABI 2012, Lecture Notes in BioInformatics 2012 7534 378 390 http://link.springer.com/chapter/10.1007/978-3-642-33122-0_30

[B7] Blanc G Ogata H Robert C Reductive genome evolution from the mother of Rickettsia PLoS Genetics 2007 3 e14 10.1371/journal.pgen.0030014 17238289 PMC1779305

[B8] Braga MDV Stoye J The solution space of sorting by DCJ Journal of Computational Biology 2010 17 9 1145 1165 10.1089/cmb.2010.0109 20874401

