# Supplementary material for: Restricted DCJ-indel model: sorting linear genomes with DCJ and indels
Source: BMC Bioinformatics. 2012 Dec 19;13(Suppl 19):S14. doi: 10.1186/1471-2105-13-S19-S14 (PMC3526439; doi:10.1186/1471-2105-13-S19-S14)
Supplement: Additional file 1 [file 1471-2105-13-S19-S14-S1.PDF]

Algorithm 1 Restricted sorting of genome A into B with optimal DCJs and indels

Input: Two linear genomes A and B
Output: A restricted sequence of DCJ and indel operations sorting A into B

```
cap genomes A and B;

  [MERGING:]
r ← null;
if there is a cycle C ∈ AG(A,B) with at least 4 vertices and at least 2 runs then
    r ← run from C;
while r ≠ null do
    extract r into a cycle;   [this preserves the indel-potential of AG(A,B) according to Proposition 5]
    r ← null;
    if a circular chromosome was created then
        find a short-link (v1, v2);   [Proposition 6]
        if (v1, v2) is a gap or a compact-run then
            apply the optimal DCJ ρ(v1, v2);
        else
            let r1 be the run that would be inversely split by ρ(v1, v2);
            if ρ(v1, v2) is the first inverted-split of r1 then
                apply the optimal DCJ ρ(v1, v2);
                let r2 be the residual part of r1;
                if r2 is in a cycle with more runs then
                    r ← r2;   [extract r2 from its cycle in the next step]
            else
                  [r1 was inversely split before and is separated alone in cycle]
                find a link (x1, x2) such that x1 is a vertex created by a previous inverted-split of r1;   [Proposition 7]
                apply the optimal DCJ ρ(x1, x2);
    if r = null and there is a cycle C ∈ AG(A,B) with at least 4 vertices and at least 2 runs then
        r ← run from C;

  [ACCUMULATING: (each cycle with 4 or more vertices has at most one run)]
while there is a long-run r in AG(A,B) do
    apply an optimal DCJ accumulating the labels of two partners of r;
    if a circular chromosome was created then
        find a short-link (v1, v2);   [Proposition 6]
        if (v1, v2) is a gap or a compact-run then
            apply the optimal DCJ ρ(v1, v2);
        else
            let r1 be the run that would be inversely split by ρ(v1, v2);
            if ρ(v1, v2) is the first inverted-split of r1 then
                apply the optimal DCJ ρ(v1, v2);
            else
                  [r1 was inversely split before and is separated alone in cycle]
                find a link (x1, x2) such that x1 is a vertex created by a previous inverted-split of r1;   [Proposition 7]
                apply the optimal DCJ ρ(x1, x2);

  [DCJ-SORTING: (each remaining cycle with 4 or more vertices has at most one compact-run)]
while there is cycle C ∈ AG(A,B) with at least 4 vertices do
    extract a cycle from C, with an optimal DCJ applied on genome A;
    if a circular chromosome was created then
        find a short-link (v1, v2);   [Proposition 6]
          [at this stage this short-link is a gap or a compact-run]
        apply the optimal DCJ ρ(v1, v2);

invert all DCJs applied on genome B;
insert each B-run r before the first inverted-split of r;
move up insertions that occur in circular chromosomes;
delete all A-runs from the DCJ-sorted components;
```
